# Housing and Environmental Enrichment of the Domestic Ferret: A Multi-Sector Survey

**DOI:** 10.3390/ani12091065

**Published:** 2022-04-20

**Authors:** Alice M. M. Dancer, María Díez-León, Jennifer K. Bizley, Charlotte C. Burn

**Affiliations:** 1 Department of Pathobiology and Population Sciences, The Royal Veterinary College, Hawkshead Lane, North Mymms, Hatfield AL9 7TA, UK; mdiezleon@rvc.ac.uk (M.D.-L.); cburn@rvc.ac.uk (C.C.B.); 2 Faculty of Brain Sciences, UCL Ear Institute, University College London, 332 Gray’s Inn Road, London WC1X 8EE, UK; j.bizley@ucl.ac.uk

**Keywords:** animal welfare, environmental enrichment, ferrets, housing, injury, questionnaire, veterinary

## Abstract

**Simple Summary:**

How ferrets across sectors are housed and the environmental enrichment provided (e.g., toys, beds, exploration of new sights and smells) can directly impact their health and wellbeing. Through an online questionnaire reaching ferret caretakers from pet owner, laboratory, zoo, rescue, and working (e.g., pest control) sectors, we describe how ferrets are housed, the enrichment they receive, enrichment types that ferrets most enjoy and those which may be harmful or problematic. Of 754 responses, 82.4% were from pet owners. Most ferrets were housed with at least one other ferret, and the type of housing varied across sectors from single-level cages to free-ranging housing. Environmental enrichments most commonly reported were hammocks, tunnels and human interaction, with ferrets reported to most enjoy digging, tunnels, human interaction and exploration. Scent trails were also reported to be among the most enjoyable enrichments but were rarely provided, suggesting that they could be used more. Problematic enrichment included rubber items, such as Kongs^®^, which could be chewed and swallowed, narrow tunnels trapping ferrets, and fabrics catching claws. These items should therefore be avoided. Our results suggest that all sectors have room to improve both housing and enrichment to better ferret welfare.

**Abstract:**

Ferrets (*Mustela putorius furo*) are kept and used in multiple sectors of society, but little is known about how they are housed and what environmental enrichment (EE) they may benefit from. We aimed to help guide caretakers about what housing and EE can be provided for ferrets. Through an online questionnaire of ferret caretakers, including pet, laboratory, zoological collection, rescue and working animal sectors internationally, we described ferret housing, opportunities for exploration, EE provision and caretaker opinions on ferrets’ preferred EE types, and problematic EE. In total, 754 valid responses from 17 countries were analysed, with most (82.4%) coming from pet owners. Most ferrets were housed socially, with housing varying across sectors from single-level cages to free-range housing in a room or outdoor enclosure; pet owners mostly used multi-level cages. The most commonly reported EE included hammocks, tunnels and tactile interaction with caretakers. Respondents reported that ferrets particularly enjoyed digging substrates, tunnels, human interaction and exploration. The most frequently reported problems were that ingestion of unsuitable chew toys and rubber items could cause internal blockages, narrow tunnels could trap ferrets, and certain fabrics that could catch claws. This suggests a need for increased awareness of the risks of these EE types and for more commercially available safety-tested ferret EE. Scent trails were relatively rarely provided but were reported to be enjoyed and harmless, so we recommend that these should be provided more commonly. Our results suggest that there is scope to improve ferret housing and EE provision to benefit ferret welfare across all sectors.

## 1. Introduction

Environmental enrichment (EE) can be defined as enhancements to an animal’s environment or husbandry to enable the opportunity for diverse behavioural repertoires and promotion of both physiological and psychological wellbeing [1,2,3]. EE can thus be highly beneficial, but inappropriate attempts at EE can be problematic, being at best ineffective and at worst stressful or injurious to the animals (e.g., [4,5,6]). There has been limited research into how ferrets (*Mustela putorius furo*) are housed and the effect of different housing types and EE on ferret behaviour. Previous questionnaire-based studies of pet ferret housing indicate that restricted space, limited out-of-house exploration time and low EE provision is associated with abnormal behaviour or biting [7,8]. Ferrets have strong preferences, as measured by their willingness to push increasingly weighted doors, for certain EE. Such experiments reveal that laboratory ferrets value restful EE (hammock or rigid cave EE), water bowls allowing for play and more natural drinking than a water nipple, conspecific companionship, foraging EE and tunnels, in that order, compared with a chamber empty of EE [9]. In a questionnaire-based study, 573 ferret pet owners reported that the EE types most commonly provided were water bowls, EE promoting rest (e.g., soft bed or hammock), tunnels and balls [7], indicating some alignment between pet owner EE provision and ferret EE preference in experimental settings. Ferrets display agonistic (combative or aggressive) behaviours toward conspecifics significantly more when in standard housing (floor substrate, a flexible plastic bucket on its side for a shelter, and food and a water bottle) and play significantly less than when housed with their preferred EE types [10]. Moreover, laboratory-housed ferrets have shown a reduction in boredom-like behaviour, such as lying awake, screeching and stimulus seeking, up to at least 24 h after receiving additional out-of-cage exploration opportunities with access to EE [11]. While these studies have provided some insight into ferret housing and EE provision, particularly in the pet and laboratory sector, we know little about both in any other ferret caretaker sectors.

Ferrets are kept by humans for a variety of purposes across many sectors of society, and we aimed to capture the variation in housing across sectors to encourage the cross-fertilisation of ideas and best practice. With an estimated 501,000 pet ferrets in the USA [12] and an estimated 1,000,000 pet ferrets in the UK [13], they are popular pets. Ferrets are also used in research and kept in laboratories, for example, 2016 newly recruited ferrets were used for scientific purposes in the EU in 2017 [14], with the total number kept for scientific purposes including breeding and non-regulated research likely to be higher still. Indeed, the number of ferrets used in research may have seen an increase in the wake of the COVID-19 pandemic, with ferrets used as a model species in SARS-CoV-2 research (e.g., [15,16]). Ferrets are also kept as working animals for hunting and pest control [17], are a popular species in zoological collections [18] and are inevitably kept in rescue centres [19].

Different sectors of animal usage differ in motivation or indeed legislation to provide EE for captive animals [20]. UK zoo licensing guidance requires extensive EE provision for all species, such as substrates, vegetation, nesting materials and burrows [21]. The UK’s code of practice for animals used for scientific practices is less explicit but requires that animals are given EE to allow them to have choice and control within their environment and to allow expression of normal behaviour [22]. However, for sectors such as pet ownership or working animals, there are no licencing bodies or formal guidelines beyond the Animal Welfare Act 2006 [23] for how ferrets should be housed and whether they should be provided with EE. Furthermore, not all UK pet owners are familiar with the Animal Welfare Act 2006, with a quarter of dog, cat and rabbit owners (in a 2018 survey of 36,494 dog, cat and rabbit owners) stating that they had not heard of the legislation [24]. Additionally, legislation to provide EE for captive animals across all sectors will differ between countries.

Some within-sector guidance on appropriate ferret housing and EE does already exist. Handbooks [25,26] on ferret care that are targeted for laboratory staff emphasise ferrets’ inquisitive, playful and exploratory natures, as well as their requirement for complex housing. They recommend EE such as tunnels, boxes, paper bags and bedding substrate to allow for tunnelling, plus space to allow ferrets to have distinct sleeping, larder, exploration or play, and latrine areas [25,26]. A minimum cage size of 0.45 m^2^ (excluding for adult entire males and adult females with young) is highlighted in Hubrecht and Kirkwood [25]. The NC3Rs guidance on ferret housing and husbandry specifies ferrets’ need for exploration and exercise, so regular out-of-cage opportunities should be provided [26]. Similar advice is available for pet owners; the RSPCA’s online ferret handbook also recommends social housing for ferrets but states that some individuals will prefer to live alone, and that groups should be compatibility matched and should not exceed four individuals [27]. The RSPCA handbook does not specify a minimum housing size, but rather sufficient space to allow ferrets to play and exercise, including daily exercise opportunities in a ferret-safe area. Additionally, some specific EE is recommended: hammocks, safe toys, tunnels, beds, water to swim in, balls, squeaky toys and digging substrates [27].

The ferret’s ideal housing and EE could be informed by the species’ natural ecology. Little is known of ferret behaviour outside captivity, because, as a domesticated species, our understanding of their natural behaviour comes largely from studies of small feral ferret populations and from the ferrets’ wild ancestor, the European polecat (*Mustela putorius*) [28,29]. Both European polecats [28] and feral ferrets [29] are predominantly solitary and live in burrows. The natural history of the European polecat can help inform EE provision for ferrets, including toys which allow motivated behaviours, such as hunting, particularly those which stimulate the olfactory or auditory senses, or those that mimic burrows through digging or burrowing substrates, tunnels and enclosed, dark sleeping areas.

The aim of the current study was to improve understanding of ferret housing and EE provision across sectors to help ferret caretakers make informed decisions about their own ferret housing and EE provision. To achieve our aim, we used an international online questionnaire to: (a) describe types of housing and out-of-house exploration opportunities provided by different sectors; (b) describe different types of enrichment used by ferret caretakers; and (c) describe which types of enrichment are deemed most enjoyable for ferrets, and which types of enrichment should be avoided. Our aim was not to focus on comparing practices between sectors, because we acknowledge that different sectors have different drivers and constraints, but we wanted to describe sector differences to help facilitate cross-sector knowledge exchange.

## 2. Materials and Methods

We conducted an online survey using SurveyGizmo^TM^ (Alchemer, Louisville, CO, USA) from 13 February to 21 March 2020. The survey was in English and open to any English-speaking participants. Members of ferret-holding laboratories who were personal contacts of the authors, as well as UK ferret-holding zoos and UK ferret rescue organisations, were contacted directly via email requesting participation. A British and Irish Association of Zoos and Aquariums (BIAZA) letter of research support was included in zoo email communication. To reach non-UK zoos, the survey was posted in Facebook^TM^ (Meta Platforms Inc., Menlo Park, CA, USA) closed zookeeper community groups. The survey was also posted in Facebook^TM^ groups related to ferret owners, working ferrets and ferret rescue organisations, and as a public post on the LinkedIn accounts of some of the authors. UK ferreting clubs were contacted directly requesting survey sharing among their memberships. Members of other relevant organisations, such as the UK’s RSPCA and the NC3Rs, were also contacted in case they could help distribute the survey. The survey received ethical approval from the Royal Veterinary College (URN SR2019-0441).

### 2.1. Survey Structure

The survey can be seen in full in the Supplementary Materials. The introductory page informed respondents that the survey’s aim was to gather information on ferret caretakers’ perception of ferrets’ needs and preferences in contribution to research into how ferrets are kept and respond to their environment. Respondents were informed the survey was anonymous, how long the survey would normally take to complete, that they must be 18 years old or over to participate and that in participating they consented for the data to be used in this research.

The survey took approximately 15 min to complete and comprised 36 questions divided into three sections. The first section asked questions pertaining to demographics, the second, housing and enrichment provision, and the third, perceptions of ferret affective states (this third section is described elsewhere [30]). Only one question, which asked respondent age, was compulsory, to allow us to ascertain that all respondents were 18 years or over. A mixture of multiple-choice, short answer and long answer formats were used. Where an ‘other’ option was selected for a multiple-choice answer, respondents were asked to specify in a long answer box. Before releasing the survey, we piloted it with individuals from the laboratory, pet owner and zoo sectors to garner feedback on question wording and the functionality of the survey software and refined the survey accordingly.

### 2.2. Survey Section One: Demographics, Respondents and Ferrets

Questions included respondent gender, age, years’ experience with ferrets, main ferret caretaker role (pet owner, working animals, laboratory, zoo, rescue, other), country of residence (if UK, respondents were asked to specify England, Northern Ireland, Scotland or Wales) and the number of ferrets currently in their care.

### 2.3. Survey Section Two: Ferret Housing and Enrichment Provision

Questions included whether ferrets were housed individually or socially, location of housing (inside, outside or access to both) and the type and complexity of housing (e.g., cage, hutch or free-range within a whole enclosure; single- or multi-level accommodation). Respondents were asked how many times per week ferrets were let out of housing and, if so, for how long. They were asked what types of EE they provided inside and outside of housing by selecting all that applied from a list; the options were randomly ordered by the SurveyGizmo^TM^ software (Alchemer, Louisville, CO, USA) for each respondent to minimise order effects (the order of questions or answers can influence how respondents answer e.g., [31]). An ‘other’ option was also available, with a free text box for details. Respondents were also asked how often EE items offered were changed. Long answer (free text) format questions were asked about which EE items ferrets seemed to enjoy the most and what led respondents to think this was the case. Finally, a long answer question requested details about whether respondents had encountered any problems (e.g., injury) with any EE items.

### 2.4. Data Cleaning and Analysis

Data from respondents who selected that they were under 18, had never cared for ferrets, or did not currently care for ferrets were removed from analysis. Respondents who left the primary caretaker role question blank were also removed from analysis, as were all surveys labelled partially completed by the SurveyGizmo^TM^ software, indicating respondents abandoned the survey part way through, or did not click through to the end of the survey.

Most results were reported as percentages of responses or other summary measures as appropriate (e.g., counts). Several questions contained answer categories from which respondents could select all which applied to them, resulting in these questions having a higher number of responses than the number of respondents. Descriptive statistics were conducted in the R software environment [32]. When several mutually exclusive answers were selected for certain multiple-choice questions (such as, ‘how many times a day do you feed your ferrets?’), answers were averaged, and categories were re-defined or lumped to create new categories to obtain one answer per respondent. Similarly, for answers to the short answer free-text question ‘How much time do they usually spend out of the hutch/cage/enclosure when let out?’ if a range was provided, the middle value was used, and all answers were re-allocated to time-period categories. For all multiple-choice questions where respondents selected ‘other’ and elucidated in the ‘please specify’ box, answers were re-allocated to one of the provided answer categories where this was reasonable (e.g., an answer of ‘taken for walks’ would be re-allocated to the existing answer category ‘Exploration of new areas’).

Reflexive thematic analysis using an inductive approach [33] (carried out by AD) was used to analyse long-form answers pertaining to ferrets’ preferred EE, why caretakers thought this to be the case, and which EE they thought was not suitable or safe for ferrets. For example, a partial respondent answer as to why they believed a certain EE to be their ferrets’ most enjoyed was “When in the ball pit they are constantly dooking”, so this was coded as “dooking” and placed into the theme “vocalisations”. To help ensure that EE prevalence did not overly bias respondents’ suggestions of which EEs were most enjoyable or problematic for ferrets, we calculated an approximate correction for this. First, we used thematic analysis to group the long answers into either the existing EE categories from our multiple-choice list or into new categories. This gave the total number of respondents who had suggested each EE type as particularly enjoyable or problematic. To ensure that the most numerous categories were not commonly suggested solely because they were also commonly provided, we then divided the number of respondents suggesting the EE type by the number of respondents who reported providing the same EE type. This could only be calculated for the EE types captured within our multiple-choice list, because we had no systematic information about how commonly other EE types were provided.

To help describe differences in EE provision between sectors of ferret use, we con-ducted a Kruskal–Wallis test of the number of EE types provided by each respondent in each sector with post hoc pairwise Dunn Tests with *p*-value adjustment using the Benjamini–Hochberg method [34] to identify where significant differences lay. The statistical significance was set at *p* = 0.05.

## 3. Results

### 3.1. Demographics

A total of 831 respondents completed the survey. Seventy-seven respondents were excluded, leaving 754 valid respondents. As only one question was compulsory, the number of respondents providing data varied by question.

Respondents represented eight sectors with pet owner being the most common (82.4%, *n* = 621), followed by zoo (6.1%, *n* = 46), rescue (5.0%, *n* = 38), working animal (3.7%, *n* = 28), laboratory (1.5%, *n* = 11) and ‘other’ (1.3%, *n* = 10).

There were 5 times more female (82.2%) than male (16.6%) respondents, 1.1% of respondents were other gender, and 0.1% of respondents did not answer. This ratio was broadly mirrored by the pet owner (female = 85.7%, male = 13.5%, other = 0.8%), zoo (f = 82.6%, m = 13.0%, o = 2.2%) and rescue sectors (f = 86.8%, m = 10.5%, o = 2.6%). However, the working animal (f = 21.4%, m = 75.0%, o = 3.6%) and laboratory (f = 36.4%, m = 63.6%) sectors had predominantly male respondents. Respondent age was mostly distributed across the youngest four age categories (18–25: 18.3%; 26–35: 30.2%; 36–45: 23.5%; 46–55: 17.4%), with few respondents aged over 56. The youngest sector was zoo (18–35: 86.9%), and the oldest sector was working animals (over 56: 32.1%).

Respondents resided in 17 countries, across six continents. Most respondents resided in England (54.5%), followed by the USA (22.1%), Scotland (8.2%), Wales (4.8%), Australia (4.2%), and Canada (1.1%).

Most respondents had 1–5 years’ experience caring for ferrets (36.9%). Pet owners had the highest proportion of respondents with 12 months or less experience (14.8%), while rescue (55.3%) and working animal (64.3%) sectors had the highest proportion of respondents with over 10 years’ experience.

The number of ferrets cared for varied by sector. Caring for 3–6 ferrets was most common overall (39.1%), and this was true for pet owners (38.8%), zoos (50%), working animals (53.6%) and laboratories (54.5%). Single ferrets were reported predominantly by pet owners (13.8%). Caring for 11–50 ferrets was most common for rescues (52.6%). There was roughly a 50:50 split between female (48.85%) and male ferrets (50.53%) across all sectors combined, with only 0.62% of ferrets of unknown sex.

### 3.2. Housing

#### 3.2.1. Individual or Social Housing

For the question of whether respondents’ ferrets were housed individually or socially, respondents could select all answer categories which applied to them. The category ‘more than two ferrets housed socially’ was the most common housing for all sectors (56.0% overall; Table 1). All sectors reported having singly housed ferrets, except laboratories, with the highest proportion found in the rescue sector (39.5%) (Table 1). Some respondents provided additional comments about their singly housed ferrets: single housing of entire males or females either during or outside of breeding season was mentioned by 14 respondents, while individual ferrets ‘preferring living alone’ was mentioned by 5 respondents.

#### 3.2.2. Housing Type

**Figure 1:**
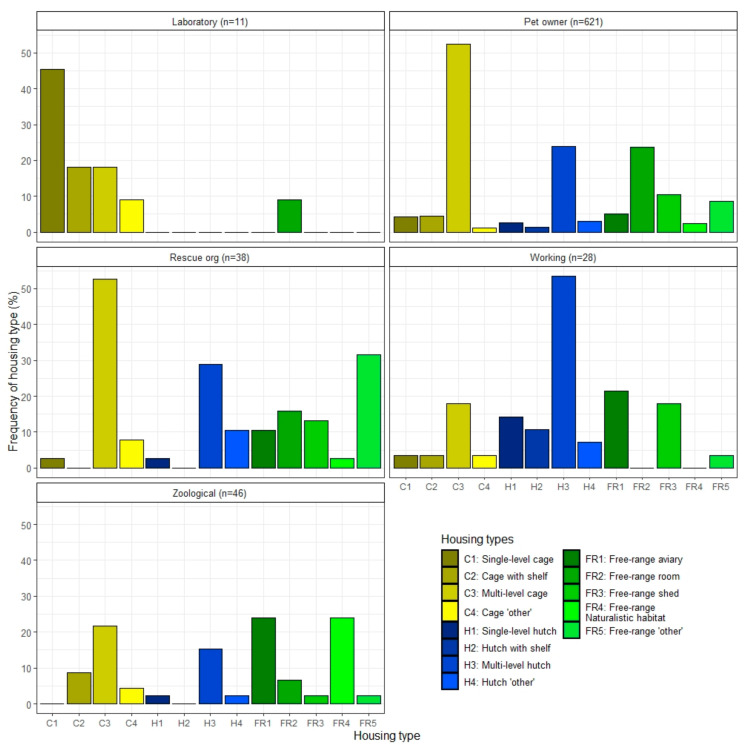
Percentage of housing types and sub-types by ferret caretaker sector. Housing types include cages (yellows), hutches (blues), and free-ranging (greens). Respondents could select all housing types which applied to them, resulting in a higher number of responses than the number of respondents.

Cages were the most common housing type for ferrets in both the pet owner (62.5%) and laboratory sectors (90.9%), hutches for the working animal sector (85.7%) and free-ranging within a room or enclosure for the rescue (73.7%) and zoo sectors (71.7%) (Figure 1). The most common cage type was multi-level for all sectors, except laboratory, where single level with no shelf was most common. A multi-level hutch was the most commonly used hutch type for all sectors, except laboratory, where hutches were never reported. Free-range housing was provided by some respondents across all five sectors, with housing ferrets free-ranging in a room being the most common free-range housing reported by the laboratory and pet owner sectors, and free-ranging in an aviary most commonly reported by the working animal and zoological sectors.

#### 3.2.3. Time Outside of Housing

All sectors reported allowing ferrets daily time outside of housing with the frequency and length of time allowed out varying within and across sectors (Table 2). The most common frequency for pet owners, rescue centres and zoos was daily, whereas for laboratories and the working sector it was 2–6 times per week. Duration of out-of-house exploration time varied within and between sectors (Table 2).

### 3.3. Environmental Enrichment

#### 3.3.1. EE Provision Inside Housing

**Figure 2:**
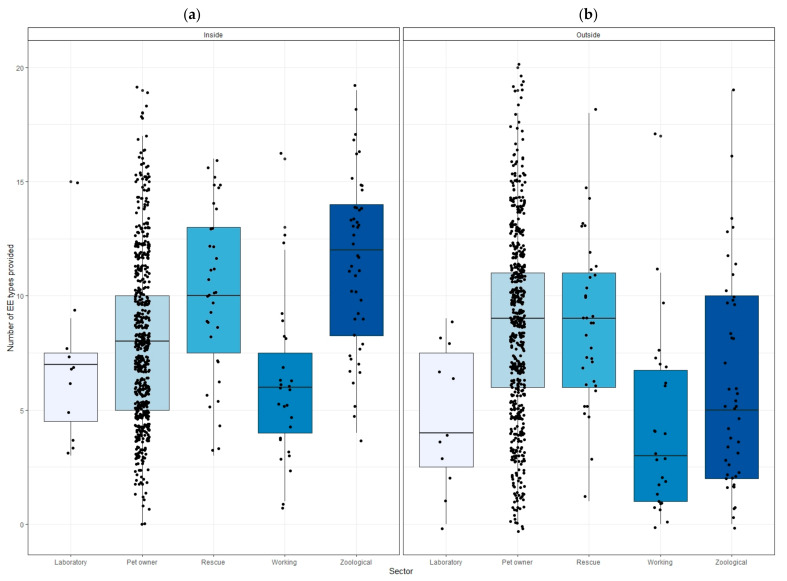
Number of enrichment types reported by caretakers to be provided inside (**a**) and outside of ferret housing (**b**) by sector. Boxes show median and first and third quartiles.

The number of enrichment types provided varied within and between sectors (Figure 2). Zoos provided the highest median number of EE types inside housing (median (IQR) = 12 (8.3–14)), and the working sector provided the least (6 (4–7.5)), with an overall median of 8 (5–11) with all sectors combined. The number of EE types provided inside housing differed significantly between sectors (Kruskal-Wallis: H4 = 51.146; *p* < 0.001).

Post hoc pairwise Dunn Tests showed significant differences, with the zoo sector providing significantly more indoor EE types than the laboratory (*Z* = −3.62, *p* < 0.001), pet (*Z* = −5.49, *p* < 0.001) and working (*Z* = −5.74, *p* < 0.001) sectors. The rescue sector provided significantly more EE types than the laboratory (*Z* = −2.71, *p* = 0.009), pet owner (*Z* = −3.23, *p* = 0.002) and working (*Z* = 4.35, *p* < 0.001) sectors, and the pet sector provided significantly more types of EE than the working sector (*Z* = 2.81, *p* = 0.008).

The most commonly reported EE types provided inside ferret housing with all sectors combined were hammocks (84.9%), tunnels (69.8%), climbing frames (66.8%), nesting materials (60.1%), bedding to cover the floor of the house (56.2%) and cat toys (50.9%). Hammocks were most frequently reported by laboratory (90.9%), pet owner (87.1%) and rescue (86.8%) sectors. Tunnels were reported most frequently by zoos (91.3%) and were the second most reported EE type by the pet owner (67.8%) and rescue (81.6%) sectors. Bedding to cover the floor was the most frequently reported EE type by the working animal (85.7%) sector (Table 3).

#### 3.3.2. EE Provision Outside of Housing

The highest median number of EE types provided outside of housing were by the pet owner and rescue (both 9 (6–11)) sectors, followed by the zoo (5 (2–10)), laboratory (4 (2.5–7.5)) and working (3 (1–6.8)) sectors, with an overall median of 8 (5–11) with all sectors combined. The number of EE types provided outside housing also differed significantly between sectors (Kruskal–Wallis: H4 = 47.418; *p* < 0.001). Post hoc pairwise Dunn Tests showed that pet owners provided significantly more EE types than the laboratory (*Z* = −3.08, *p* = 0.005), working (*Z* = 5.08, *p* < 0.001) and zoo (*Z* = 3.90, *p* = 0.0003) sectors. The rescue sector also provided significantly more types of EE outside of ferret housing than the laboratory (*Z* = 2.80, *p* = 0.008), working (*Z* = 4.04, *p* < 0.001) and zoo (*Z* = 2.81, *p* = 0.009) sectors.

Tunnels (74.4%), tactile interaction with caretaker (72.1%), boxes (67.4%) and exploration of new areas (66.0%) were the most reported EE types provided outside of ferret housing with all sectors combined, and this was broadly mirrored within sectors (Table 3). The most reported EE types provided outside housing differed from EE provided inside housing, which had higher numbers of certain restful EE (hammocks, nesting materials), while tunnels were highly reported both inside and outside of ferret housing. Where respondents selected ‘other’ for EE either inside or outside of ferret housing, their corresponding long answers were extremely varied (Table 4).

#### 3.3.3. Frequency of EE Change

**Figure 3:**
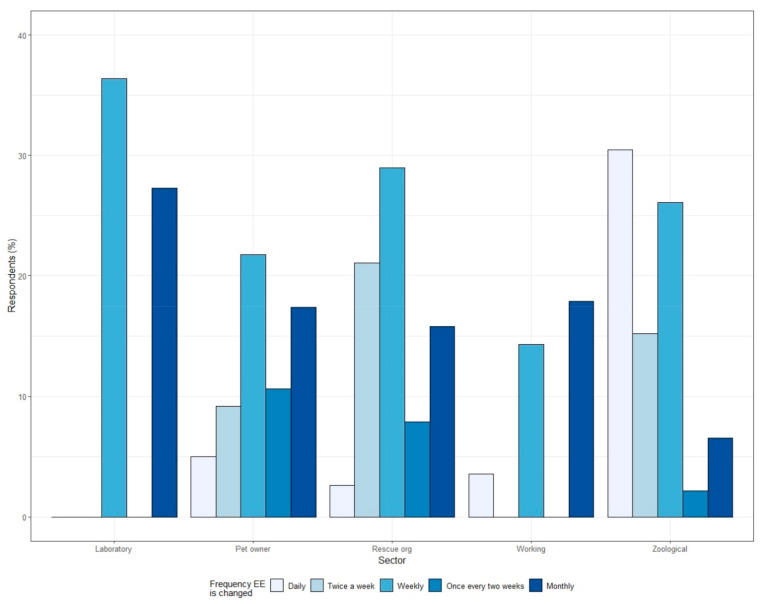
Frequency that respondents from different caretaker sectors reported changing EE items provided to their ferrets.

Changing EE weekly (22%) was the most common frequency for all sectors combined, followed by monthly (17%) changes of EE. Weekly and monthly EE changes were most common for the laboratory, pet and working sectors, while changing EE either twice a week or weekly was most common for the rescue sector, and daily and weekly changes most common for the zoological sector (Figure 3). However, a high proportion of respondents chose not to answer this question (laboratory = 36.4%, pet owner = 39.5%, rescue = 31.6%, working = 67.9%, zoo = 21.7%).

#### 3.3.4. Enjoyable EE

**Figure 4:**
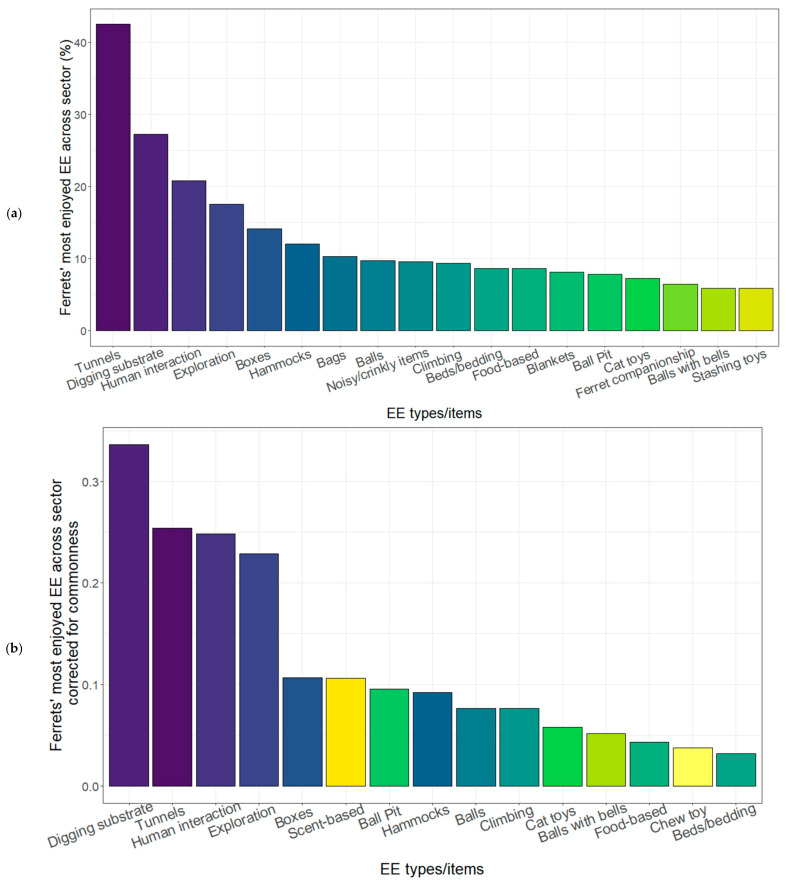
Caretakers’ opinions of ferrets’ most enjoyed EE types. In (**a**), the data are organised in decreasing order of the raw percentage of respondents suggesting the EE most enjoyed by their ferrets. Only the EE types that were reported by ≥5% of respondents are shown. In (**b**), caretakers’ opinions of ferrets’ most enjoyed EE types when corrected for commonness of provision of EE type are shown. Only EE types listed in the multiple-choice list for question ‘which EE types do you offer your ferret’ could be corrected for commonness. For both (**a**,**b**), the purple to yellow colour gradation indicates most to fewer absolute numbers of respondents reporting the EE to be enjoyed. EE types suggested by fewer than 5% of respondents as ferrets most enjoyed EE types, and therefore not shown, were water (4.9%), soft toys (4.8%), toys on a string/stick (4.5%), dog toys (2.5%), scent-based EE (1.8%), shoes (1.8%), socks (1.5%), grass (1.4%), chew toys (1.4%) and rocks/logs (0.5%).

A total of 649 respondents provided long-format answers regarding their opinion of the EE types that ferrets most enjoy. The most enjoyable EEs were considered to be tunnels (42.5%), digging substrates (27.3%), human interaction (20.8%) and exploration (17.6%) (Figure 4). Moreover, worth highlighting were bags and noisy EE, which were reported as enjoyed by about 10% of respondents, despite us having omitted them from our multiple-choice list (Figure 4a), and scent-based EE, which were frequently reported as enjoyed even though they were rarely provided (Figure 4b). Although hammocks and climbing frames were among the five most commonly provided EEs for ferrets, they were not among the top five enjoyed EE items.

Long-format answers were provided by 448 respondents as to why they thought the above items were their ferrets’ most enjoyed EE items, and 50.4% reported that the vocalisation ‘dooking’ (also called ‘chuckling’ or ‘clucking’) while interacting with the EE was indicative of enjoyment. The ‘weasel war dance’ (also known as ‘dance of joy’, ‘joy jumps’, or ‘Freudensprünge’) behaviour was also frequently stated as an indicator of enjoyment (25.9%), as was running or running backwards (14.3%), high frequency of choosing an item (12.7%), and jumping (12.5%) and bouncing (11.8%) behaviours.

#### 3.3.5. Problematic EE

Long format answers were given by 297 respondents listing EE types with which they had experienced problems (Table 5). Problems ranged from ferrets avoiding certain items through to fatalities caused by strangulation or internal blockages. The most commonly reported problems were ingestion causing internal blockages (45.1%) and the claws or other body parts becoming trapped (28.6%). Of the EE types that we listed as multiple-choice options, those of most concern included a variety of chew toys and puzzle feeders, such as Kong^®^ toys (The Kong Company, Salisbury, Wilts, UK), that caused problems when ingested, and tunnels that were too narrow or made of materials that could trap ferrets. Items that did not fit into our suggested EE types followed a similar theme, with respondents commonly raising concerns about ferrets ingesting rubber items and having claws or other body parts caught and injured by fabric bedding. Running wheels were frequently cautioned against, given how rarely provided they were, but the absolute number of respondents providing running wheels was small (only 14). On the other hand, the safest EE types, which were provided by over 10% of respondents and yet never reported to be problematic, were exploration, buried/scattered food, scent trails and sounds or music.

## 4. Discussion

This survey shows there to be great diversity in the provision of ferret housing, out-of-house exploration opportunities and EE provision, both between and within different ferret care-taking sectors. This indicates that there is room to improve housing and EE provision within and across sectors on a case-by-case basis. Where EE is provided, there does appear to be some consensus as to EE types which are most enjoyed by ferrets and EE types which may need to be used with caution. We will discuss each of these aspects in turn.

### 4.1. Housing

#### 4.1.1. Individual or Social Housing

Social housing of ferrets in groups of more than two was the most commonly reported housing for all sectors (52–84% of respondents, depending on the sector). Group housing allows for ferrets to participate in positive social interactions, and ferrets continue to participate in social play with conspecifics into adulthood [35]. However, the ferrets’ wild ancestor, the European polecat, is a solitary species, with intraspecific tolerance only observed in the first year between juveniles of the same litter and between males and females during the breeding season [28]. It, therefore, may not be surprising to find ferrets who prefer solitary living, and care should be taken to observe for individual preferences (aggression between ferrets or withdrawn behaviour could indicate an unsuitable social grouping [35]). It has been recommended that ferret social groupings should not exceed four individuals [35], as aggression can occur. Indeed, high numbers of ferrets kept together, or non-compatible individuals housed together, can lead to chronic stress resulting in common ferret illnesses associated with *Helicobacter* infections, such as gastric ulcers [35,36]. Our findings suggest the social needs of most ferrets are likely being met, although certain individuals may be at risk of chronic stress when housed in groups. Some respondents reported they housed intact animals alone outside of the breeding season; this practice could be of welfare concern where individuals who would rather live with a companion are being housed individually. The laboratory sector was the only sector not to report housing any ferrets individually, which is in line with the UK national guidance on the use of animals in research [22], although the number of laboratory respondents was low, so may not represent the whole sector. The large number of ferrets kept in laboratories may allow this sector to more easily house compatible individuals together, yet this sector may be housing certain ferrets socially who would prefer to be housed alone.

#### 4.1.2. Housing Type

The reported housing of 9.4% of ferrets in single-level cages/hutches, with or without a shelf, is of welfare concern. Housing type, in terms of the space and its complexity, available to the ferrets, can potentially impact their welfare. Insufficient enclosure space can result in both physiological [37,38] and psychological (e.g., [39]) changes in captive species. For example, body size, specifically ulna and tibia bone development, has been found to be altered by small enclosure size in a closely related species, the black-footed ferret (*Mustela nigripes*) [37]. The tibias and ulnas were up to 9% shorter in black-footed ferrets housed in small enclosures (1.2 × 2.4 × 0.9 m), whereas larger enclosure sizes (6 × 6 × 3.6 m) did not show any differences in tibia length to wild-living counterparts. The home range size of the ferret’s closest wild counterparts, the European polecat, is an average of 181 ha [40], and the home range of feral ferrets in New Zealand is 12.4 ha for females and 31.4 ha for males [29], so it would not be surprising if housing in the suggested minimum cage size for one to two ferrets of 1.5–2 m^2^ [35] would lead to welfare issues, such as abnormal behaviour [41] or boredom [42]. Discouragingly, commercially available ferret cages often do not meet this suggested minimum cage size e.g., ferret cages sold by two UK pet stores at the time of writing [43,44] had a floor space of just 0.4 m^2^ (single level cage) and 1.21 m^2^ (multi-level cage), respectively. This has the potential to be very misleading to ferret caretakers looking to buy suitable ferret housing and makes it of even greater welfare concern for ferrets who do not receive regular out-of-cage exploration if housed in these small commercially available houses. The laboratory sector reported the highest use of single-level cages with or without a shelf, while both single-level cages and single-level hutches with a shelf were most frequently used by the working ferret sector. Importantly, free-ranging housing was represented within all sectors, if rarely in the small sample of laboratory sector respondents in this study.

Free-ranging housing, if fully fitted with furniture (e.g., tunnels, hides, beds, dig pits), can provide highly complex environments, while still allowing space for multiple and varied EE items. Reports from a ferret pet-owner survey (respondent *n* = 573) indicate that ferrets housed free-ranging in a dedicated room or in the entire home showed significantly less stereotypical pacing and repetitive nibbling than ferrets housed inside an indoor enclosure (cage, hutch or other modified space, e.g., wardrobe) [7]. The reporting of free-ranging housing across sectors is certainly a positive sign for ferret welfare.

#### 4.1.3. Time Outside of Housing

Nearly all respondents reported providing exploration time outside of enclosures, with only 9.1% of laboratories and 1.1% of pet owners never providing this opportunity. Providing time to explore outside could be an extremely valuable tool in enhancing ferret welfare. Exploration time, especially in a space with EE, allows exercise and mental stimulation and has been found to reduce the signs of boredom in laboratory-housed ferrets as much as 24 h after returning to their home cage [11]. Exploration time could be particularly vital to those ferrets housed in the more restrictive housing types, such as the single-level cages and hutches. It is important to recognise that allowing ferrets out-of-housing opportunities to explore and play should only be provided supervised in a ferret-safe zone away from potential hazards or areas of escape, such as in a dedicated ferret-proofed room or in a play-pen type area, especially as ferrets are notorious for ingesting inappropriate items [45,46]. Moreover, it is important to ensure that the ferrets’ vaccinations are up to date if the exploration opportunities are provided outside [35].

### 4.2. Environmental Enrichment

#### 4.2.1. EE Provision Inside and Outside Housing

The five EE types reportedly most enjoyable to ferrets (tunnels, digging substrate, tactile interaction with a caretaker, exploration of new areas, and boxes) aligned closely with the most reported EE types provided outside ferret housing (tunnels, tactile interaction with a caretaker, exploration of new areas, and boxes), rather than those provided inside ferret housing. Only tunnels were in the top five reported EE types provided inside ferret housing. Providing more ‘enjoyable’ EE within housing could, where practical (e.g., boxes and digging substrate), elevate ferret welfare.

Within all sectors combined, an average of 8 (5–11) EE types were provided, yet some respondents from all sectors reported providing high numbers of EE types inside housing (Figure 2). Behavioural indicators of positive welfare, specifically the ‘dooking’ or ‘chuckling’ vocalisation and the ‘weasel war dance’ or ‘joy jumps’ behaviour, have been seen to increase with the number of EE provided [8], suggesting that more EE types is better for welfare. In contrast, two respondents from the pet owner sector reported never providing any of the listed EE types inside ferret housing, and indeed all sectors had low minimum numbers of EE types provided inside housing. The low average for both the working animal and the laboratory sectors, when viewed alongside their high reporting of housing in single-level hutches and cages, indicates that these animals may be less likely to have opportunities to experience positive welfare than those in other sectors. However, working-sector ferrets hunt prey, which is likely to be highly enriching, both through fulfilling their behavioural drive for prey and through their exposure to multi-sensory novelty (e.g., following scent trails, digging, tactile interaction with caretakers) with each new hunt location. Indeed, laboratory ferrets prefer contrafreeloading (choosing to work for food even when food is freely available), pushing weighted doors to access forage balls which allow ferrets to mimic the behaviours of hunting prey [9]; they also show reduced signs of boredom up to 24 h after being allowed to play outside their home-cages [11]. Therefore, if ferrets in the working sector are taken hunting regularly, this could mitigate the effects of lower EE provision through exposure to diverse sensory stimulation alongside satisfying the high behavioural motivation to hunt (albeit only appetitive behaviour, as hunting ferrets do not consume their prey). It is possible that more restrictive housing types do not offer the space necessary to provide a larger number of EE types, in which case the refinement of EE provision through regular rotation of stimulating EE to reduce habituation and promote interest for the ferrets could be beneficial [47]. The number of out-of-housing opportunities ferrets receive can also interact with the number of EE types provided. For example, ferrets who have less time out-of-housing and who receive fewer EE types bite caretakers or conspecifics causing injury more frequently [8].

#### 4.2.2. Frequency of EE Change

For EE that is intended to stimulate (rather than relax) ferrets, habituation can reduce its effectiveness, and, in a review of this topic, Tarou and Bashaw [47] identified that behaviour towards EE decreases both within a session of the EE being presented and between multiple sessions, even if the EE contains a positive reinforcer, such as food. The authors suggested rotating a large number of different items in an unpredictable order to minimise habituation to EE. Consequently, respondents who reported changing EE with only a monthly frequency are potentially reducing the effectiveness of their EE and creating a monotonous and unstimulating environment, risking boredom [11,48]. One caveat is that ferrets may benefit from less regular changing of EE that is intended to promote rest, sleep or refuge, which may provide security in their permanence [49]. A limitation of the current study is the high rate of respondents who chose not to answer this question, which may have resulted in the rarer answers being missed.

#### 4.2.3. Enjoyable EE

Caretakers from across sectors believed their ferrets enjoy digging substrate, tunnels, human interaction, exploration, boxes and scent-based EE, in that order, when responses were corrected for commonness. This ranking does not completely align with ferret choices in a formal preference test; in a seven-chamber experimental set-up, laboratory ferrets worked hardest (pushing a weighted door) to reach restful EE, particularly hammocks, followed by water bowls, social EE, foraging opportunities and tunnels, respectively [9]. Perhaps most notably, hammocks and social interaction with other ferrets were not listed amongst the most enjoyable EE in our questionnaire. One reason for the discrepancy will be that only a subset of EE types could be tested in the seven-chamber study, compared with this questionnaire, and yet some EE that Reijgwart et al. [9] tested was rarely provided by respondents (e.g., water bowls for paddling and foraging opportunities). Moreover, Reijgwart et al.’s [9] measure of preference was the amount of weight pushed to gain access, whereas we asked respondents, most of whom were pet owners, about what their ferrets ‘enjoyed’ most, which is subtly different from ‘preference’. Caretakers in the current study reported that they identified ferret enjoyment of EE both via behaviours indicative of excitement or play, such as ‘dooking’, ‘weasel war dance’ and ‘running’, but also via the ferrets frequently choosing to interact with the EE type. While this latter sign could encompass not only EE types which promote play or other activity, but also restful and sleep behaviours, it is possible that caretakers may have understood the question of ‘enjoyment’ as their ferrets actively enjoying the EE, and so inactive enjoyment of restful EE, such as hammocks, may have been selected by respondents less readily. Providing ferrets with preferred types of EE can promote signs of positive welfare in experimental settings, with ferrets housed with their preferred EE types (hammocks, forage ball and water bowl) performing increased social play compared to ferrets housed with unpreferred EE types (ferret ball and golf ball) or no EE (only a shelter and floor substrate) [10]. A future consumer demand study using EE types suggested by caretakers as being enjoyed by ferrets would be useful for industry sectors that may require greater evidence-based support of new EE provision. Additionally, a future questionnaire enquiring about ferrets’ most enjoyed EE at the individual level could strengthen our understanding of EE most enjoyed by ferrets when housed in the same conditions (in multi-ferret settings) and potentially illuminate ferret-personality differences in EE preferences. In the meantime, we recommend providing ferrets with the stimulating EE that ferrets are reported to enjoy here alongside the hammocks and (where possible) paddling water that they have been shown to work hard to access [9].

Caretakers also listed a wide range of other types of EE which they provided for their ferrets, from different types of digging substrate, to different spices and scent ideas for sensory enrichment. This list is a useful resource for caretakers from any ferret caretaker sector who might be seeking inspiration for different types of EE to provide for their ferrets. This study indicates that scent-based EE is underused as a form of EE for ferrets, ranking highly in the commonness-corrected list of enjoyed EE, whilst being reported as a type of EE offered relatively rarely (Table 3). EE that stimulate the olfactory sense are behaviourally relevant to a ferret’s natural history, as odours are important, both through olfactory communication between ferrets and through scent marking of valued resources, such as food sources [50]. Scent-based stimuli can be beneficial for animal welfare, especially when provided in a manner that allows voluntary interaction, so that the animal can choose whether to approach or avoid the stimulus [3]. In rodents, some specific scents have been found to be anxiolytic [51], while others can be anxiogenic [52], but it is not known which scents should be recommended for ferrets, and this is worth investigating further. Peppermint and bitter apple scents should be avoided, unless intended as a deterrent to chewing furniture [11]. As well as scents, paper bags and noisy or crinkly items could be a useful EE to provide, as both ranked highly as enjoyable EE, despite not being in the multiple-choice answer list.

#### 4.2.4. Problematic EE

Caretakers provided detailed lists of EE items with which they had had negative experiences, either through ferret illness or injury, or simply because their ferrets ignored the items. Although this list is anecdotal and personal to each of the respondents, some items were repeatedly suggested as being problematic. Some of the highest reported problematic EE types were items made of rubber: chew toys (often rubber or similar material); puzzle feeders, especially Kong^®^ toys; and general reference to ‘rubber’ items, which is supported by veterinary opinion and case reports of intestinal blockages caused by ferrets ingesting rubber in the literature (e.g., [35,45,46,53]). Tunnels were also highly reported as problematic for two main reasons. Firstly, tunnels which were too narrow resulted in ferrets becoming trapped. Ensuring that tunnels are wide enough for ferrets to pass through and turn around in will be important to minimise this risk. Secondly, tunnels made of certain (usually unspecified) fabrics resulted in ferrets catching their claws and injuring themselves. Similarly, soft beds and fabric bedding materials were highly reported, as they also caused ferrets to catch their claws, suggesting preventative claw trimming to be an important regular husbandry procedure. It will be necessary to identify which types of fabric pose less of a risk, and thus should be used in commercially available ferret EE. Some types of balls were mentioned as causing problems, such as rubber balls and tennis balls. Consequently, any interaction with EE types not specifically designed for ferrets should be closely supervised, and all EE items should be regularly checked for wear and tear. Commercially available ferret enrichment should be rigorously safety tested, if it is not already.

## 5. Conclusions

While ferrets are kept and used across many different sectors, this study has described variation in ferret housing and EE provision within and between sectors. Our results show that larger, more complex housing, extended opportunities for out-of-housing exploration and greater EE provision is possible within all sectors, as demonstrated by those respondents who are providing all of these elements for their ferrets. We recommend ferret caretakers move away from housing ferrets in single-level cages or hutches and provide regular out-of-house exploration opportunities in ferret-safe environments to avoid detrimental impacts to welfare. Indeed, we would recommend a revision of existing guidelines to remove the use of single-level cages or hutches for ferrets, except in extenuating circumstances, e.g., medical or age-related reasons. Further, we recommend providing a large number of different EE types to promote opportunities for positive welfare, particularly the preferred EE types identified here: digging substrates, tunnels, human interaction, exploration, boxes and scent-based EE. We also recommend a further study to explore whether the EE types identified as most enjoyed by ferrets here are also those which ferrets seem to enjoy the most in an experimental setting, including both stimulating and restful EE. Caretakers should avoid providing—or allowing ferrets access to—rubber items, narrow tunnels or items made of fabric that could catch claws. These findings can be used to enable caretakers to make positive steps towards improvement in their housing or EE provision in every sector. Our study provides useful information on the reported EE preferences of ferrets, inspiration for EE types to provide and useful advice for caretakers on EE types which may cause harm to their ferrets.

## Figures and Tables

**Table 1:** Ferret housing status (individually or socially housed) broken down by sector. Respondents could select all applicable categories, resulting in a higher number of responses than the number of respondents.

Ferret Social Housing Status	Laboratory (*n* = 11)	Pet Owner (*n* = 621)	Rescue (*n* = 38)	Working (*n* = 28)	Zoological (*n* = 46)	Total (*n* = 744)
On their own (individually)	0.0%	24.3%	39.5%	17.9%	13.0%	23.8%
In pairs	36.4%	35.1%	39.5%	28.6%	39.1%	35.3%
More than two housed socially	72.7%	52.8%	84.2%	67.9%	65.2%	56.0%
Mother with kits	9.1%	0.6%	7.9%	3.6%	2.2%	1.3%
Unanswered/left blank	0.0%	0.5%	2.6%	3.6%	0.0%	0.7%

**Table 2:** Out-of-house exploration opportunities provided to ferrets by caretaker sector.

		Respondents % (*n*)				
Weekly Frequency Ferrets Let Out of Enclosure	Length of Time Spent Out of Enclosure	Laboratory (*n* = 11)	Pet Owner (*n* = 621)	Rescue (*n* = 38)	Working (*n* = 28)	Zoological (*n* = 46)
Daily	All durations	36.4 (4/11)	75.2 (467/621)	71.1 (27/38)	39.3 (11/28)	41.3 (19/46)
	≤30 min	25.0 (1)	1.9 (9)	3.7 (1)	18.2 (2)	26.3 (5)
	31 min to 1 h	25.0 (1)	8.1 (38)	14.8 (4)	36.4 (4)	10.5 (2)
	1 h 1 min to 2 h	0.0	19.9 (93)	29.6 (8)	0.0	0.0
	2 h 1 min to 4 h	0.0	27.8 (130)	18.5 (5)	18.2 (2)	36.8 (7)
	>4 h	25.0 (1)	34.3 (160)	11.1 (3)	27.3 (3)	0.0
	Not applicable	0.0	0.2 (1)	3.7 (1)	0.0	0.0
	Blank/missing data	25.0 (1)	7.7 (36)	18.5 (5)	0.0	26.3 (5)
2 to 6 times a week	All durations	45.5 (5/11)	11.4 (71/621)	10.5 (4/38)	42.9 (12/28)	30.4 (14/46)
	≤30 min	20.0 (1)	9.9 (7)	75.0 (3)	16.7 (2)	50.0 (7)
	31 min to 1 h	20.0 (1)	15.5 (11)	0.0	16.7 (2)	21.4 (3)
	1 h 1 min to 2 h	60.0 (3)	32.4 (23)	0.0	33.3 (4)	14.3 (2)
	2 h 1 min to 4 h	0.0	25.4 (18)	0.0	8.3 (1)	7.1 (1)
	>4 h	0.0	5.6 (4)	25.0 (1)	8.3 (1)	7.1 (1)
	Not applicable	0.0	1.4 (1)	0.0	8.3 (1)	0.0
	Blank/missing data	0.0	9.9 (7)	0.0	8.3 (1)	0.0
Once a week or varying	All durations	9.1 (1/11)	2.6 (16/621)	2.6 (1/38)	10.7 (3/28)	15.2 (7/46)
	≤30 min	0.0	18.8 (3)	0.0	0.0	14.3 (1)
	31 min to 1 h	0.0	25.0 (4)	0.0	33.3 (1)	57.1 (4)
	1 h 1 min to 2 h	0.0	25.0 (4)	100.0 (1)	0.0	14.3 (1)
	2 h 1 min to 4 h	100.0 (1)	25.0 (4)	0.0	0.0	0.0
	>4 h	0.0	6.3 (1)	0.0	66.7 (2)	14.3 (1)
	Not applicable	0.0	0.0	0.0	0.0	0.0
	Blank/missing data	0.0	0.0	0.0	0.0	0.0
Never		9.1 (1/11)	1.1 (7/621)	0.0	0.0	0.0
Unanswered/missing data		0.0	9.7 (60/621)	15.8 (6/38)	7.1 (2/28)	13.0 (6/46)

**Table 3:** Percentage of respondents providing each EE type listed in a multiple-choice answer, either inside ferret housing (columns headed ‘In’), or outside ferret housing (columns headed ‘Out’) during out-of-cage exploration time. Respondents could select all EE types they provide, so the number of responses exceeds the number of respondents. EE types are listed in alphabetical order.

EE Type Location	EE Type	Respondents Providing EE Type Inside/Outside Housing (%)									
		Laboratory		Pet Owner		Rescue		Working		Zoological	
		In	Out	In	Out	In	Out	In	Out	In	Out
EE types listed as multiple-choice options.	Ball pits	9.1	27.3	22.7	48.8	42.1	50	3.6	7.1	54.3	34.8
	Balls	63.6	54.5	46.4	67.3	60.5	57.9	25.0	14.3	60.9	37.0
	Balls with bells in	36.4	36.4	44.9	57.8	63.2	60.5	3.6	0	54.3	13.0
	Bedding to cover the floor	54.5	9.1	54.1	14.7	52.6	18.4	85.7	21.4	67.4	19.6
	Boxes	36.4	54.5	44.1	71.5	68.4	65.8	28.6	21.4	82.6	43.5
	Buried/scattered food	0	18.2	18.0	16.4	21.1	13.2	10.7	10.7	45.7	17.4
	Cat toys	27.3	18.2	53.3	64.1	55.3	50	10.7	0	43.5	19.6
	Caves to sleep under	54.5	0	43.3	32.5	57.9	36.8	32.1	10.7	63.0	13.0
	Chew toys	45.5	18.2	14.7	18.4	13.2	15.8	10.7	10.7	17.4	4.3
	Climbing frame	36.4	27.3	66.8	40.7	68.4	44.7	50.0	21.4	80.4	32.6
	Different flavours of food	18.2	27.3	37.0	22.4	39.5	7.9	39.3	28.6	39.1	10.9
	Different textures of food	27.3	9.1	33.5	15.9	34.2	2.6	35.7	10.7	43.5	4.3
	Exploration of new areas	n/a	45.5	n/a	67.0	n/a	63.2	n/a	46.4	n/a	73.9
	Food in puzzle feeders	9.1	0	12.6	14.3	21.1	13.2	7.1	3.6	47.8	21.7
	Hammocks to sleep in	90.9	9.1	87.1	28.8	86.8	34.2	46.4	17.9	73.9	13.0
	Nesting materials	63.6	0	58.5	26.4	57.9	15.8	78.6	25.0	78.3	19.6
	Running wheel	0	0	0.6	1.0	0	0	3.6	0	2.2	2.2
	Scent trails	0	9.1	4.2	6.9	5.3	7.9	7.1	10.7	45.7	26.1
	Sounds or music	0	9.1	2.7	8.1	10.5	13.2	3.6	3.6	23.9	6.5
	Substrate to dig in	18.2	0	27.1	40.9	28.9	44.7	14.3	25.0	69.6	47.8
	Tactile interaction with caretaker	n/a	36.4	n/a	74.2	n/a	81.6	n/a	35.7	n/a	71.7
	Tunnels	54.5	63.6	67.8	77.5	81.6	81.6	64.3	46.4	91.3	52.2
	Other	0	0	10.0	15.3	28.9	23.7	7.1	17.9	8.7	15.2
	None of the above	0	0	0.6	0.2	2.6	0	0	3.6	0	2.2
	Not applicable	n/a	9.1	n/a	1.1	n/a	0	n/a	7.1	n/a	2.2
EE types not listed as multiple-choice but regularly provided as an ‘other’ EE type.	Blankets	0	n/a	5.6	n/a	2.6	n/a	0	n/a	0	n/a
	Soft toys	0	0	2.1	1.0	0	0	0	3.6	0	0
	Rope toys	27.3	n/a	28.0	n/a	31.6	n/a	14.3	n/a	50.0	n/a
	Water	n/a	0	n/a	1.8	n/a	10.5	n/a	7.1	n/a	0

n/a indicates not applicable.

**Table 4:** Descriptions of EE items provided by respondents in the long answer format ‘other EE type’ answer box from across sectors.

EE Type	EE Item Detail Suggested by Three or More Respondents (*n*)
Dig box substrate EE	Leaf litter (3)
	Packing peanuts (3)
	Shredded paper (3)
	Soil (4)
	Water (25)
Sleeping EE	Cave/soft cat bed (8)
	Dog bed (3)
	Hanging basket (4)
	Hanging nesting/sleep pouch/tube/cube (5)
	Hide box (3)
	Wicker basket (3)
Foraging/Food EE	Carcass feed of fresh game meat (3)
	Fish oil (3)
	Training session (3)
Exploration/Sensory EE	Enclosed garden access (8)
	Walks to the park/woodland/fields/outside (16)
Toys/play EE	Baby toys (rattle/soft cubes/hanging, crinkly and squeaky elements) (8)
	Bags (paper/fabric/plastic if supervised) (17)
	Branches (3)
	Bubble wrap/bubble shipping envelope (3)
	Cloth tug toy (3)
	Crawling inside furniture (9)
	Crinkle bag/tube/ball/toys (11)
	Artificial grass (4)
	Interaction with other pets (cats/dogs) (7)
	Large logs to climb on (6)
	Old shoes/boots (without laces) (4)
	Old/worn clothing/sheets (7)
	Paddling/swimming pool (10)
	Rocks (3)
	Scratching post (5)
	Socks (3)
Scent trail EE: Spices and extracts	Herbs (7)
	Perfume/ aftershave (9)
	Spices (10)
	Vanilla (4)
Scent trail EE: Animal scents	Access to recently vacated other animal enclosures (4)
	Animal bedding (e.g., rabbit/rat) (9)
	Animal fur/wool (e.g., rabbit/sheep/alpaca) (9)
	Feathers (5)
	Fish oil (7)
	Items (e.g., tunnels/boxes) used by prey species (rabbits) (3)
Scent trail EE: Other scents	Hidden treats (5)
	Plants (8)
	Sticks from woodland (5)

**Table 5:** Environmental enrichment (EE) types which caretakers reported as being problematic, either due to risk to ferrets, ferrets ignoring or rarely using the EE, or due to the EE being quickly and easily destroyed (financially impractical). EE types are listed in order from EE type with the highest approximate rate of reported problems to the fewest. Reasons given for the EE type being problematic are listed with the number of respondents reporting the reason shown in brackets. If any specific types of EE were mentioned in relation to the EE type these are also listed alongside the number of respondents reporting the item in brackets.

EE Class Status	EE Type	Number of People Reporting Problems	Number of People Providing EE Type	Approximate Rate of Reported Problems	Reported Reasons for Problem (*n*)	Specific Forms of EE Type Mentioned (*n*)
EE listed as multiple-choice options and reported as problematic	Running wheel *	2	14	0.143	Ignore (1); Damage back (1)	n/a
	Chew toys	20	241	0.083	Ingestion and internal blockage (16); Ignore/avoids (2); Choke risk (1); Teeth caught (1)	Dog tyre chew toy (1); Hard plastic (1); Rawhide (1); Rubber chew toys (1); Soft plastic (1); Soft rubber toys (1); Soft squeaky puppy toy (1)
	Food in puzzle feeders	10	221	0.045	Ingestion and internal blockage (8); Aggression (1); Ignore/avoids (1)	Kong^®^ (8); Puzzle/ball feeder (2)
	Tunnels/tubes	31	1087	0.029	Trapped (13); Strangulation risk (5); Claws caught (4); Injury (4); Ignore (2); Broke quickly (1); Ingestion and internal blockage (1); Death (1)	Fabric tunnel (10); Narrow tunnels such as toilet or kitchen roll tubes (10); Tunnels with metal wires (3); Tunnel with lining (1); Crinkle tunnel (1); ‘Marshall Super Thru-Way’ tunnel (1)
	Rope toys	6	219	0.027	Ingestion and internal blockage (3); Claws caught (1); Ignored (1); Unspecified (1)	Rope ladder (1)
	Digging substrate	14	526	0.027	Ingestion and internal blockage (7); Skin/eye irritation (2); Bored/ignore (1); Defecation (1); Ignore/avoids (1); Respiratory problems (1); Sand temperature too cold (1)	Sand (4); Rice (3); Packing peanuts (2); Pasta (2); Shredded paper (1); Bean bag foam balls (1); Sawdust (1)
	Climbing frame	20	803	0.025	Injury (14); Escape (1); Ignore/avoids (1); Trapped (1); Penis caught (1)	Ladder (4); Cat tower/tree (3); Ramp (1); Cage shelf (1)
	Balls	20	828	0.024	Ingestion and internal blockage (15); Choke risk (2); Ignore/avoids (2); Teeth caught (1)	Rubber balls (6); Tennis balls (6); Balls (non-specified) (3); Balls with holes (1); Cat balls (1); Foam balls (1); Ping-pong balls (1); Small balls (1)
	Cat toys	19	816	0.023	Ingestion and internal blockage (7); Ignore/avoids (3); Choke risk (2); Injury (2); Claws caught (1); Strangulation risk (1); Teeth caught (1); Trapped (1); Unspecified (1)	Cat toys on a string (7); Foam cat toys (2); Toy mice (2); Feather toys (2); Cat toys (unspecified) (2); Soft cat toys (1); Cat toys that can unravel (1); Cat toys with small holes (1); Catnip toys (1)
	Different textures of food	5	367	0.014	Ignore/avoids (2); Risk of insulinoma (1); Choke risk (1); Ingestion and internal blockage (1)	Novel food (3); Freeze dried meat (1); Whole prey carcass (1)
EE class status	EE type	Number of people reporting problems	Number of people providing EE type	Approximate rate of reported problems	Reported reasons for problem (*n*)	Specific forms of EE type mentioned (*n*)
	Ball Pit	7	534	0.013	Ingestion and internal blockage (2); Ignore/avoids (2); Respiratory problems (1); Strong aversion (lunge/bite/flee) (1); Injury if damaged (1)	n/a
	Hammocks	11	848	0.013	Claws caught (8); Destroys (1); Ingestion and internal blockage (1); Strangulation risk (1)	Damaged hammocks (6); Hammocks made from loose material (1)
	Balls with bells in	9	734	0.012	Ingestion and internal blockage (4); Teeth caught (3); Destroys (2); Ignore/avoids (1)	n/a
	Bedding to cover floor	6	541	0.011	Respiratory problems (3); Ingestion and internal blockage (2); Ignore/avoids (1)	Sawdust/shavings (4); Carefresh’ bedding (1); Critter bedding (1)
	Different flavours of food	3	440	0.007	Ignore/avoids (2); Risk of insulinoma (1)	Novel food (3)
	Caves to sleep under	2	566	0.004	Ignore/avoids (1); Trapped (1)	Cave bed (1); Wooden bird nest box (1)
	Nesting materials	2	640	0.003	Claws caught (1); Respiratory problems (1)	Hay (1); Wool type bedding (1)
	Boxes	2	863	0.002	Penis caught (1); Ignore/avoids (1)	n/a
	Human interaction	1	544	0.002	injury (1)	n/a
EE not listed as multiple-choice options and reported as problematic	Soft beds and fabric bedding materials	31	n/a	n/a	Claws caught (17); Strangulation risk (6); Ingestion and internal blockage (5); Injury (2); Trapped (1)	some bedding’ (7); blankets (4); ripped/damaged bedding (3); soft beds (3); towels (2); materials bedding’ (2); fleece (2); beds with satin type lining (2); hide-n-sleep bed (2); fluffy cat bed (1); clothes (1); loose weave scarfs (1); labels on bedding/beds (1);
	Rubber items	29	n/a	n/a	Ingestion and internal blockage (27); *Unspecified* (2)	n/a
	Damaged toys	14	n/a	n/a	Injury (7); Ingestion and internal blockage (5); Claws caught (1); Teeth caught (1)	n/a
	Soft toys	14	n/a	n/a	Ingestion and internal blockage (13); Ignore/avoids (1)	n/a
	Squeaker toys	11	n/a	n/a	Strong aversion (lunge/bite/flee) (7); Ingestion and internal blockage (3); Unspecified (1)	n/a
	All toys if not rotated	8	n/a	n/a	Bored/ignore (8)	n/a
EE class status	EE type	Number of people reporting problems	Number of people providing EE type	Approximate rate of reported problems	Reported reasons for problem (*n*)	Specific forms of EE type mentioned (*n*)
‘Safe’ EE: frequently selected from multiple-choice options, but not reported as problematic	Exploration	0	498	0.000	n/a	n/a
	Buried/scattered food	0	268	0.000	n/a	n/a
	Scent trails	0	113	0.000	n/a	n/a
	Sounds or music	0	97	0.000	n/a	n/a

* The approximate rate of the reported problem for running wheels must be treated with caution as very few respondents reported providing this EE type. n/a indicates not applicable.

## Data Availability

The data presented in this study are available on request from the corresponding author. The data are not publicly available due to participant consent to their data being used for research only of the nature described in the questionnaire introduction.

## Supplementary Materials

The following supporting information can be downloaded at: https://www.mdpi.com/article/10.3390/ani12091065/s1, File S1: Ferrets: A survey of housing, management and welfare.
