# Correction: Dancer et al. Housing and Environmental Enrichment of the Domestic Ferret: A Multi-Sector Survey. *Animals* 2022, *12*, 1065

**DOI:** 10.3390/ani12223160

**Published:** 2022-11-16

**Authors:** Alice M. M. Dancer, María Díez-León, Jennifer K. Bizley, Charlotte C. Burn

**Affiliations:** 1 Department of Pathobiology and Population Sciences, The Royal Veterinary College, Hawkshead Lane, North Mymms, Hatfield AL9 7TA, UK; 2 Faculty of Brain Sciences, UCL Ear Institute, University College London, 332 Gray’s Inn Road, London WC1X 8EE, UK

## Text Correction

There was an error in the original publication [1]. We mistakenly reported that there was an “…estimated 1,000,000 pet ferrets in the UK [13]”. This should have read as an “…estimated 100,000 pet ferrets in the UK [13]”.

A correction was implemented in Section 1 in the second paragraph of the Introduction:

“…an estimated 100,000 pet ferrets in the UK [13]”.

The authors state that the scientific conclusions are unaffected. This correction was approved by the Academic Editor. The original publication has also been updated.

## References

[1] Dancer A.M.M., Díez-León M., Bizley J.K., Burn C.C. (2022). Housing and Environmental Enrichment of the Domestic Ferret: A Multi-Sector Survey. Animals.

