# Supplementary material for: Housing and Environmental Enrichment of the Domestic Ferret: A Multi-Sector Survey
Source: Animals (Basel). 2022 Apr 20;12(9):1065. doi: 10.3390/ani12091065 (PMC9102951; doi:10.3390/ani12091065)
Supplement: Supplementary file 1 [file animals-12-01065-s001.zip › animals-1645970-supplementary.pdf]

File S1: Ferrets: A survey of housing, management, and welfare

I am a doctoral student at the Royal Veterinary College. The purpose of this survey is to gather data on ferret caretakers' perception of ferret's needs and preferences. The data will directly contribute to our research into how ferrets are kept and how they respond to their environment.

This questionnaire is anonymous and usually takes 12 minutes. If you start it now, you can save your answers and complete it later. By participating in this survey, you consent for the data to be used in this research and any subsequent publications and presentations. Data will be stored following good research practice guidelines.

The questionnaire will be split into three parts. **Part one** asks some general questions about you and your ferrets, including in what capacity you look after ferrets. **Part two** asks how you house them. **Part three** asks what your perception is of ferret welfare needs more generally.

Pet owners and professionals are equally welcome to contribute. For those working professionally with ferrets and answering on behalf of your business/organisation please note that for the purpose of this questionnaire the ferrets will be referred to as 'your' ferrets. You must be aged 18 years or over to participate.

Alice Dancer (PhD researcher)
Contact details: adancer@rvc.ac.uk

Part one

1. What is your gender?
    a. Female
    b. Male
    c. Other
    d. Prefer not to say

2. What is your age?
    a. Under 18
    b. 18 to 25
    c. 26 to 35
    d. 36 to 45
    e. 46 to 55
    f. 56 to 65
    g. 65 and over

3. How many years' experience do you have with ferrets?
    a. Less than 1 month
    b. 1 to 12 months
    c. 1 to 5 years
    d. 6 to 10 years
    e. Over 10 years
    f. I used to care for ferrets but do not at present
    g. I have not yet cared for ferrets, but know a lot about them

4. How would you define your main role as a ferret caretaker?
    a. Pet owner
    b. Pet shop
    c. Working animals (ferreting/pest control)
    d. Breeder

e. Laboratory/Research
f. Zoological collection
g. Rescue organisation
h. Other, please state

5. Do you also additionally care for/work with ferrets in any of the following ways (tick all that apply)?
    a. No
    b. Pet owner
    c. Working animals (ferreting/pest control)
    d. Breeder
    e. Other *(please specify)*

6. In which country are you and the ferrets you care for situated (If you're in the UK please specify whether England, Northern Ireland, Scotland or Wales)?

7. How many ferrets do you currently care for?
    a. 1
    b. 2
    c. 3-6
    d. 7-10
    e. 11-50
    f. 51-100
    g. Over 100
    h. I do not currently have any ferrets

8. What sex are your ferrets? (tick all that apply)
    a. Female
    b. Male
    c. Have both female and male ferrets
    Unsure

9. What is the neuter status of your ferrets?
    a. Neutered
    b. Entire
    c. Have both neutered and entire ferrets
    d. Unsure

10. How old is/are your ferret/s? (if you have more than one please state the range)
    Drop down boxes:
    Ferret age/min age: (0-15)
    Max age (if applicable): (0-15)

Part two

1. Are your ferrets housed socially or individually?
    a. On their own (individually)
    b. In pairs
    c. More than two housed socially
    d. Mother with pups
    e. Other *(please specify)*

2. How are your ferrets housed?

    a. Inside
    b. Outside
    c. Both

3. What is their housing like? (tick all which apply) *Drop downs for the sub categories*
    a. Hutch
        i. single level
        ii. with a shelf (accessible via ramp)
        iii. multi-level
        iv. other *(please specify)*
    b. Cage
        i. single level
        ii. with a shelf (accessible via ramp)
        iii. multilevel
        iv. other *(please specify)*
    c. Free range within a whole enclosure
        i. Shed
        ii. Aviary
        iii. Zoo-style habitat enclosure
        iv. Indoor room
        v. Other *(please specify)*

4. How often are they let out of the hutch/cage/enclosure?
    a. Daily
    b. 5 to 6 times a week
    c. 2 to 4 times a week
    d. once a week
    e. less than once a week
    f. other *(please specify)*

5. How much time do they usually spend out of the hutch/cage/enclosure when let out?
    a. <15mins
    b. 15 to 30mins
    c. 31 to 1hr
    d. 1hr1min to 2hr
    e. 2hr1min to 4hr
    f. 4hr1min to 12hr
    g. >12hr
    h. Not applicable
6. How many times a day are your ferrets fed?
    a. Once a day
    b. Twice a day
    c. Three times a day
    d. Four or more times a day *(please specify)*
    e. Constant access to food

7. What sorts of things do you provide **inside** the ferret home cage/enclosure? (tick any that apply)
    a. Nesting materials
    b. Bedding to cover the floor, e.g. sawdust
    c. Caves to sleep under
    d. Hammocks to sleep in
    e. Substrate to dig in e.g. sand or earth

f. Tunnels/tubes to crawl through
g. Boxes
h. Ball pits
i. Balls
j. Balls with bells in
k. Chew toys, e.g. nylabone, raw hide
l. Cat toys e.g. toys hanging from a string
m. Food in puzzle feeders, e.g. kong, flip and slide feeder
n. Different flavours of food
o. Different textures of food
p. Buried/scattered food
q. Scent trails/things to smell (please describe below)
r. Sounds or music e.g. playing recordings of mouse calls
s. Climbing frame, e.g. ladders/ramps
t. Running wheel
u. None of the above (recognising that many things may not be practical in some settings)
v. Other *(please specify)*

8. What sorts of things do you offer **outside** their home cage/enclosure? (select as many as apply) Please note, training is not included in this list and will be considered separately later.
    a. Nesting materials
    b. Bedding to cover the floor, e.g. sawdust
    c. Caves to sleep under
    d. Hammocks to sleep in
    e. Substrate to dig in e.g. sand or earth
    f. Tunnels/tubes to crawl through
    g. Boxes
    h. Ball pits
    i. Balls
    j. Balls with bells in
    k. Chew toys, e.g. nylabone, raw hide
    l. Cat toys e.g. toys hanging from a string
    m. Food in puzzle feeders, e.g. kong, flip and slide feeder
    n. Different flavours of food
    o. Different textures of food
    p. Buried/scattered food
    q. Scent trails/things to smell (please describe below)
    r. Sounds or music e.g. playing recordings of mouse calls
    s. Climbing frame, e.g. ladders/ramps
    t. Running wheel
    u. None of the above (recognising that many things may not be practical in some settings)
    v. Not applicable
    w. Other *(please specify)*

9. Which of the above things you provide do your ferrets seem to enjoy the most?

10. Why do you think this (e.g. what behaviour do you see when they are interacting with those things)?

11. Have you encountered any problems with any things you have provided for your ferret, e.g. injuries or ferrets avoiding items?

12. How often do you change the items offered?
    a. Daily

b. Twice a week
c. Weekly
d. Once every two weeks
e. Monthly
f. Other *(please specify)*

13. For what purpose do you train your ferret(s), if at all? (Select all which apply)
    a. Basic handling (e.g. coming when called)
    b. Husbandry (e.g. crate training to take to vets, nail trimming)
    c. Work (e.g. specific trained behaviours for research or recall training during pest control excursions).
    d. Tricks (e.g. obstacle course, playing dead)
    e. I have not needed to, or been able to, start training my ferret(s) so far
    f. Other *(please specify)*

14. Below are scenarios involving different methods of training. Please indicate the likelihood you would act in the way the scenario outlines.
    a. Your ferret enters her/his carry-crate when you asked so you give her/him a reward
    Extremely likely, likely, neutral, unlikely, extremely unlikely, not applicable

    b. Your ferret bites you so you tap her/him on the nose
    Extremely likely, likely, neutral, unlikely, extremely unlikely, not applicable

    c. Your ferret starts to get over excited while you are playing with her/him, so you give her/him a 'time out' by returning her/him to its cage
    Extremely likely, likely, neutral, unlikely, extremely unlikely, not applicable

    d. You are taking your ferret for a walk on a harness. Your ferret starts to pull so you stop walking until the ferret stops pulling, then you continue walking
    Extremely likely, likely, neutral, unlikely, extremely unlikely, not applicable

    e. Your ferret jumps on a piece of furniture you don't want her/him to go on, so you shout at her/him to get off
    Extremely likely, likely, neutral, unlikely, extremely unlikely, not applicable

    f. You call your ferret to come to you and you reward her/him when it does
    Extremely likely, likely, neutral, unlikely, extremely unlikely, not applicable

    g. You are holding your ferret tightly because it is wriggling, but you relax your grip once she/he stays still
    Extremely likely, likely, neutral, unlikely, extremely unlikely, not applicable

    h. You are playing with a toy with your ferret. Your ferret starts to bite you during the play, so you remove the toy
    Extremely likely, likely, neutral, unlikely, extremely unlikely, not applicable

    i. Your ferret is digging at the floor and damaging it, so you spray her/him with some water
    Extremely likely, likely, neutral, unlikely, extremely unlikely, not applicable

j. You want to move your ferret from one part of her/his home to another. To do this you move a board slowly towards her/him until she/he moves away. Once she/he's moved away from the board you move the board back
Extremely likely, likely, neutral, unlikely, extremely unlikely, not applicable

k. Your ferret tends to bite you when you stroke her/him, so you give her/him a reward whenever she/he lets you stroke her/him without biting
Extremely likely, likely, neutral, unlikely, extremely unlikely, not applicable

Part three

1. What behaviours might you expect to see from a happy, stimulated ferret, compared with when it is awake but neutral (not happy, not sad)? Tick all that apply.

a. Sleeping more than normal (avg. 14 -18 hours a day)	n. Scratching at cage/enclosure walls	aa. Resting/sleeping huddled with cage mate.
b. Sleeping less than normal	o. Very responsive to sights and sounds	bb. **Vocalisations:**
c. Eating more, or more frequently, than normal	p. Ignoring new sights and sounds	cc. 'Dook'
d. Eating less, or less frequently, than normal	q. Focused on what it is doing	dd. 'Chuckling'
e. Self-grooming	r. Looking around as if alert to surroundings	ee. Barking
f. Grooming or playing with other ferrets	s. Yawning	ff. Scream/screeching
g. Interacting with enrichment (e.g. toys or nesting materials)	t. Resting with eyes open	gg. Hissing
h. Aggression towards other ferrets	u. **Resting body positions:**	hh. Whimpering/whining
i. Hiding	v. Resting/sleeping on back with belly exposed	ii. Being quiet
j. Pacing back and forth	w. Resting/sleeping on belly	jj. All of the above
k. Active behaviours such as running, digging, exploring	x. resting/sleeping curled up	kk. None of the above
l. Dance of joy	y. Resting/sleeping head raised	ll. Other (please specify)
m. Ferret war dance	z. Resting/sleeping head down	

2. What behaviours might you expect to see from a relaxed ferret?

a. Sleeping more than normal (avg. 14 -18 hours a day)	n. Scratching at cage/enclosure walls	aa. Resting/sleeping huddled with cage mate.
b. Sleeping less than normal	o. Very responsive to sights and sounds	bb. **Vocalisations:**
c. Eating more, or more frequently, than normal	p. Ignoring new sights and sounds	cc. 'Dook'
d. Eating less, or less frequently, than normal	q. Focused on what it is doing	dd. 'Chuckling'

e. Self-grooming	r. Looking around as if alert to surroundings	ee. Barking
f. Grooming or playing with other ferrets	s. Yawning	ff. Scream/screeching
g. Interacting with enrichment (e.g. toys or nesting materials)	t. Resting with eyes open	gg. Hissing
h. Aggression towards other ferrets	u. **Resting body positions:**	hh. Whimpering/whining
i. Hiding	v. Resting/sleeping on back with belly exposed	ii. Being quiet
j. Pacing back and forth	w. Resting/sleeping on belly	jj. All of the above
k. Active behaviours such as running, digging, exploring	x. resting/sleeping curled up	kk. None of the above
l. Dance of joy	y. Resting/sleeping head raised	ll. Other (please specify)
m. Ferret war dance	z. Resting/sleeping head down	

3. What behaviours might you expect to see from a fearful or distressed ferret?

a. Sleeping more than normal (avg. 14 -18 hours a day)	n. Scratching at cage/enclosure walls	aa. Resting/sleeping huddled with cage mate.
b. Sleeping less than normal	o. Very responsive to sights and sounds	bb. **Vocalisations:**
c. Eating more, or more frequently, than normal	p. Ignoring new sights and sounds	cc. ‘Dook'
d. Eating less, or less frequently, than normal	q. Focused on what it is doing	dd. ‘Chuckling'
e. Self-grooming	r. Looking around as if alert to surroundings	ee. Barking
f. Grooming or playing with other ferrets	s. Yawning	ff. Scream/screeching
g. Interacting with enrichment (e.g. toys or nesting materials)	t. Resting with eyes open	gg. Hissing
h. Aggression towards other ferrets	u. **Resting body positions:**	hh. Whimpering/whining
i. Hiding	v. Resting/sleeping on back with belly exposed	ii. Being quiet
j. Pacing back and forth	w. Resting/sleeping on belly	jj. All of the above
k. Active behaviours such as running, digging, exploring	x. resting/sleeping curled up	kk. None of the above
l. Dance of joy	y. Resting/sleeping head raised	ll. Other (please specify)
m. Ferret war dance	z. Resting/sleeping head down	

4. What behaviours might you expect to see from a bored ferret?

a. Sleeping more than normal (avg. 14 -18 hours a day)	n. Scratching at cage/enclosure walls	aa. Resting/sleeping huddled with cage mate.
b. Sleeping less than normal	o. Very responsive to sights and sounds	bb. **Vocalisations:**
c. Eating more, or more frequently, than normal	p. Ignoring new sights and sounds	cc. ‘Dook'

d.	Eating less, or less frequently, than normal	q.	Focused on what it is doing	dd.	'Chuckling'
e.	Self-grooming	r.	Looking around as if alert to surroundings	ee.	Barking
f.	Grooming or playing with other ferrets	s.	Yawning	ff.	Scream/screeching
g.	Interacting with enrichment (e.g. toys or nesting materials)	t.	Resting with eyes open	gg.	Hissing
h.	Aggression towards other ferrets	u.	**Resting body positions:**	hh.	Whimpering/whining
i.	Hiding	v.	Resting/sleeping on back with belly exposed	ii.	Being quiet
j.	Pacing back and forth	w.	Resting/sleeping on belly	jj.	All of the above
k.	Active behaviours such as running, digging, exploring	x.	resting/sleeping curled up	kk.	None of the above
l.	Dance of joy	y.	Resting/sleeping head raised	ll.	Other (please specify)
m.	Ferret war dance	z.	Resting/sleeping head down		

5. On average how many hours a day do you think your ferret/s is/are awake?
    a. Don't know
    b. 10 to 12
    c. 7 to 9
    d. 4 to 6
    e. 3 hours or less
    f. Other *(please specify)*
6. Do you think ferrets can experience boredom?
   Definitely, Very probably, Probably, Possibly, Probably not, Definitely not

7. Please explain why you think ferrets can or can't experience boredom

8. Do you think the ferrets you care for have ever experienced boredom?
    a. Yes
       Explain why you think this, behaviours you have seen which suggest boredom:
    b. No
       Explain why you think this:
9. How often do you think your ferret/s experience/s boredom?

Continuously, most of the time, occasionally, rarely, never

10. What do you think is necessary to prevent boredom?
    a. Social housing with other ferrets
    b. Interaction with other species (e.g. other pets or animals)
    c. Interaction with familiar humans
    d. Being outside
    e. Having time to explore outside its home cage
    f. Offering food in a bowl
    g. Offering food so the ferret must work to access it
    h. Having tunnels or nesting areas
    i. Toys in their home cage e.g. balls
    j. Nothing
    k. Other *(please specify)*

Thank you for participating in this survey.

Alice Dancer.
adancer@rvc.ac.uk
